# Effect of Combined Treatment of Heat Moisture and Ultrafine Grinding on the Quality of Gluten-Free Brown Rice Biscuits

**DOI:** 10.3390/foods14213763

**Published:** 2025-11-02

**Authors:** Shan Zhang, Di Yuan, Bin Hong, Shan Shan, Jingyi Zhang, Song Yan, Shiwei Gao, Qing Liu, Shuwen Lu, Chuanying Ren

**Affiliations:** 1 Food Processing Research Institute, Heilongjiang Academy of Agricultural Sciences, Harbin 150086, China; zhangshanfood@163.com (S.Z.); yuandi199707@163.com (D.Y.); gru.hb@163.com (B.H.); 18845896856@163.com (S.S.); 18846080235@139.com (J.Z.); bsbaobei@sina.com (S.Y.); 2 Heilongjiang Province Key Laboratory of Food Processing, Harbin 150086, China; 3 Heilongjiang Province Engineering Research Center of Whole Grain Nutritious Food, Harbin 150086, China; 4 Suihua Branch of Heilongjiang Academy Agricultural Sciences, Suihua 152001, China; gaoshiwei1118@126.com (S.G.); liuqing58627@163.com (Q.L.)

**Keywords:** heat moisture treatment, ultrafine grinding, brown rice, gluten-free biscuits, quality analysis

## Abstract

Brown rice is a nutritious, gluten-free whole grain, the edible potential of which is limited by inferior palatability and storage stability. In this study, brown rice (20% *w*/*w*) was subjected to heat moisture treatment (HMT) at 110 °C for 2 h, followed by ultrafine grinding, to prepare gluten-free biscuits, which were compared with those made from wheat flour, white rice, and brown rice. The results showed that the content of dietary fiber (2.67–3.62%), total phenolic (0.053–0.154%), and vitamin E (0.574–1.483 mg/100 g) in brown rice biscuits after combined treatment was enhanced compared with wheat flour biscuits. The spread ratio (4.06–8.89), hardness (700.82–1085.91 g), and brittleness (1068.89–2067.18 g/sec) of the biscuits were significantly improved (*p* < 0.05). Scanning electron microscopy revealed that the biscuits treated with combined treatment had fewer cavities and a more compact texture. The biscuits made from HMT brown rice demonstrated a reduced peroxide value, with a slower increase in acid value (0.19–0.21 mg/g) compared to untreated samples (0.24–0.38 mg/g) during storage. The innovative combined treatment of HMT and ultrafine grinding improved qualities of brown rice biscuits. This approach expands the utilization potential of brown rice, while also offering a viable strategy for grain conservation and loss reduction.

## 1. Introduction

Biscuits, a common bakery product, are highly favored by consumers and producers because of their portability, ready-to-eat nature, and relatively long shelf life [1]. Biscuits are mainly composed of wheat flour, oil, and sugar, with high calorie content and single nutritional composition. The amino acid composition of wheat flour is unbalanced, and moreover, wheat gluten can cause celiac disease, non-celiac gluten sensitivity, and wheat allergies [2,3]. Accordingly, there is increasing interest in the development of gluten-free biscuits [4,5].

Rice (*Oryza sativa* L.) is a widely grown crop and the main food source for billions of people worldwide [6,7]. Rice is husked to obtain brown rice, which is rich in vitamins, minerals, dietary fiber and other bioactive ingredients, which has certain advantages in antioxidation, obesity reduction, diabetes and other diseases [8,9,10]. Kalahal et al. developed a snack through the extrusion of tigernut and brown rice blends, which demonstrated not only higher protein content but also remarkable antioxidant activity [11]. Moreover, brown rice is gluten-free and less allergenic. Wei et al. applied brown rice to the production of gluten-free rice cake, which exhibited favorable sensory properties catering to the needs of the gluten-sensitive population [12]. Consequently, brown rice holds significant promise for application in the gluten-free products. However, owing to the presence of bran, brown rice grains present a larger particle size in the crushing process, resulting in a sense of granularity when eaten. Additionally, enzymes in the bran of brown rice can cause lipid hydrolysis during storage, thereby promoting the deterioration of brown rice and its products and further restricting its application.

Given this, overcome the textural and stability challenges of brown rice biscuits is crucial. Prior studies indicated that smaller particle sizes positively influence foods, including Asian noodles, snack biscuits, and flat breads [13]. Ultrafine grinding technology is an emerging grinding technique that grinds materials to micrometer, submicron, or nanometer sizes (1 nm~100 µm) while retaining the original characteristics [14]. In general, finer-sized particles more readily release flavor compounds and also greatly improve the taste of the food product [15]. Ultrafine grinding technology has been applied to grains with high dietary fiber content, such as whole wheat and black beans, with the result that active ingredients are more easily dissolved in the food product and taste is enhanced [5,16]. Furthermore, heat moisture treatment (HMT) is a stabilization method that has been extensively studied in recent years. HMT is primarily performed by conditioning grain samples to a moisture content below 35% (which is insufficient for gelatinization) and then subjecting them to elevated temperatures of 90–120 °C for a duration of 25 min to 12 h [17]. This process not only induces alterations in the crystalline structure of starch but also effectively inhibits the activity of endogenous enzymes [18,19]. As HMT requires only thermal energy and water, it is regarded as a simple, efficient, and relatively environmentally friendly technique [20]. Previous studies have shown that HMT not only enhances some of the nutrients in the raw ingredients of noodles and other foods but also has a beneficial effect on the quality of their derived products [21]. However, extensive research has focused on the effects of HMT on the physicochemical and digestive properties of rice flour or starch, while research on its impact on the nutritional and storage quality of brown rice and its products is scarce [17].

In this study, a combination of HMT and ultrafine grinding was used to process brown rice for the production of gluten-free biscuits. The differences in nutritional composition, physical properties, textural properties, microstructure, sensory evaluation, and storage properties of three tested grain products—biscuits made with either ultrafine grinding white rice, brown rice, or heat moisture treated brown rice—were compared with each other and with the control material (biscuits made with wheat flour). The quantitative and qualitative (microstructure imaging) data support the feasibility of preparing gluten-free biscuits from brown rice treated with heat moisture and ultrafine grinding and serve as a reference for more intensive processing of brown rice.

## 2. Materials and Methods

### 2.1. Materials

The ingredients for the wheat biscuits, namely wheat flour and butter, were purchased from a local market. Brown rice was provided by the Suihua Branch of Heilongjiang Academy of Agricultural Sciences. The brown rice was then milled using a rice polisher (VP-32, Yamamoto, Onomichi, Japan) to remove the bran layer through the method of sand roller friction, obtaining white rice samples. All chemical reagents were of analytical grade.

### 2.2. Heat Moisture Treatment (HMT)

200 g of brown rice (i.e., hulled rice with bran intact) were moisture-conditioned by adding deionized water to a constant level of 20%, followed by equilibrating in a refrigerator at 4 °C for 24 h. The sample was then placed in a sealed container and transferred to a hot-air oven(DHG-9013A, Yiheng Technical Co., Ltd., Shanghai, China) and heated at 110 °C for 2 h, after which the rice was dried at 40 °C for 24 h to reach the initial moisture content of the rice (9.2%, *w*/*w*), and place it in a sealed bag for later use [22]. This treatment is a type of physical modification of starch and enzymes to stabilize brown rice, reduce rancidity, and improve flour functionality for biscuits.

### 2.3. Ultrafine Grinding and Preparation of Biscuits

Brown rice, white rice, and HMT brown rice were crushed using an ultrafine grinder (LWF-12B1, Jinan Longwei, Jinan, China) at a speed of 920 rpm for 5 min to obtain ultrafine crushed particles, which were sealed and stored at 4 °C for future use.

Biscuits were prepared using the method described by Islam et al. with slight modifications [23]. With 100 g of wheat flour as the benchmark, 30 g of butter, 15 g of sugar, 15 g of whole milk powder, 0.4 g of salt, 2 g of baking powder, and 35 g of whole milk were added, respectively, to form a dough. The dough was pressed into a 4 mm thick layer in a round mold (5 cm diameter) and baked in a baking oven for 15 min at 160–165 °C. All biscuit formulations shared identical ingredients, with the only variation being the primary raw material (i.e., the flour type). The biscuits prepared from 100% wheat flour, 100% white rice flour (ultrafine grinding), 100% brown rice flour (ultrafine grinding) and 100% heat moisture treated brown rice flour (ultrafine grinding) were labeled control, WRB, BRB and HBRB, respectively.

### 2.4. Proximate Composition Analysis of Biscuits

Moisture, ash, total protein, fat, and total dietary fiber were all determined according to the standard of the American Association of Cereal Chemists, with total dietary fiber assessed specifically by the enzymatic–gravimetric procedure [24]. The carbohydrate was calculated using the difference method. The total phenol content (TPC) was measured by the Folin–Ciocalteu method [25]. Vitamin B_1_ and B_2_ contents were determined by high-performance liquid chromatography (HPLC) with fluorescence detection following acid extraction and enzymatic digestion. Vitamin E (as α-tocopherol) was analyzed by HPLC following alkaline saponification and solvent extraction [26].

### 2.5. Analysis of the Diameter of Biscuits

A Vernier caliper (JS20, Shengtaixin Electronic Technology Co., Ltd., Huzhou, China) was used to determine the diameter and thickness (in mm) of the biscuits. The diffusion ratio of the biscuits was determined by dividing the diameter by the thickness [27].

### 2.6. Color Analysis

The color of biscuits was measured using a colorimeter (CR-400, Konica Minolta, Tokyo, Japan) in the CIE system (L*, a*, b*). In this analysis, L* value is a measure of brightness, the values of a* and b* represent redness and yellowness, respectively. Each parameter was determined in triplicate. The ΔE value was calculated based on the color values of the control biscuit [28].

### 2.7. Textural Properties

Textural properties of the baked biscuits, such as hardness and brittleness, were determined using TAXT-Plus (TA.XT. plus, Stable Micro Systems Ltd., Godalming, UK). The test was carried out using a P/2 probe, and a force was applied at the center of the biscuit, with a pre-test speed of 1.0 mm/s, a mid-test speed of 1.0 mm/s, a post test speed of 10.0 mm/s, a test displacement of 2.5 mm, and a trigger load of 20.0 g. The sample was chosen randomly, six times per batch [29].

### 2.8. Sensory Evaluation Analysis

Sensory evaluation testing was conducted with reference to the method of Yang et al. with some modifications [30]. A panel of twenty consumers (aged 25–55 years) was recruited. It should be noted that, due to resource constraints in the initial project phase, this sample size (*n* = 20) is below the ideal number recommended by ISO 11136:2014 [31], which constitutes a limitation of the experimental method. Therefore, this study should be considered exploratory, and the findings will require confirmation through larger-scale validation studies. Prior to formal evaluation, all participants attended an orientation session to familiarize themselves with the testing procedure and the use of the 9-point hedonic scale (where 9 = extremely liked, 8 = liked very much, 7 = moderately liked, 6 = slightly liked, 5 = neither liked nor disliked, 4 = slightly disliked, 3 = moderately disliked, 2 = disliked very much, and 1 = extremely disliked) for expressing their personal preference. Sensory quality characteristics were then evaluated using this scale. The appearance, color, aroma, texture (explained as hardness and brittleness), and mouthfeel of the biscuits were evaluated. Between evaluations, purified water was used as a flavor neutralizer by the panelists.

### 2.9. Scanning Electron Microscope (SEM)

The microstructure of the biscuit was determined by scanning electron microscopy (SEM, QUANTA200, FEI Company, Hillsboro, OR, USA) using the method described by Wang et al. with slight modifications [32]. The samples were carefully cut into small pieces and fixed on an aluminum workbench using double-sided tape, followed by sputter-coating with a thin layer of gold. The samples were then imaged at an accelerating voltage of 20 kV and a magnification of 500×.

### 2.10. Storage Properties

The baked biscuits were let to cool and packaged in sealed polypropylene plastic, then placed in a 40 °C constant temperature cabinet for accelerated testing. The storage period was 7 weeks, and samples were collected every 7 days to test for moisture, peroxide value, and free fatty acids.

Acid value was determined according to the AOAC method [24]. Briefly, the extracted oil was dissolved in a mixture of petroleum ether and ethanol (1:1, *v*/*v*) and titrated with 0.1 N potassium hydroxide (KOH) solution to a potentiometric endpoint. Peroxide value was analyzed based on the method described by Kumar. et al. The oil sample was dissolved in a chloroform–glacial acetic acid mixture (3:2, *v*/*v*). Saturated potassium iodide (KI) solution was then added, and the liberated iodine was titrated with 0.002 N sodium thiosulfate (Na_2_S_2_O_3_) standard solution for calculation [33].

### 2.11. Statistical Analysis

Each experiment was individually carried out with at least three replications, and the obtained results were expressed as the mean ± standard deviation. ANOVA and Duncan’s multiple range test (*p* < 0.05) were carried out using SPSS 23.0 software. Origin 2021 software was used for plotting.

## 3. Results

### 3.1. Nutritional Analysis

In Table 1, we present a comprehensive evaluation of the nutritional composition of the different biscuits. With the exception of protein and moisture, which can be attributed to the inherent composition of the raw materials, the BRB and HBRB had higher contents of fat, dietary fiber, total phenolics, vitamins, and ash than the control and WRB. In particular, VB_1_ and VB_2_ contents were not detected in the control and WRB, whereas the VB_1_ and VB_2_ contents in BRB and HBRB were 0.065–0.069 mg/100 g and 0.126–0.179 mg/100 g, respectively. This is mainly because brown rice bran is rich in bioactive substances, such as dietary fiber, total phenols, VB_1_ and VB_2_, and mineral components. The embryo of a brown rice grain is rich in fat and vitamin E; therefore, the nutritional content of the two groups containing brown rice was higher than that of the control and WRB samples [34,35]. Simultaneously, HMT leads to a reduction in the content of certain heat-sensitive nutrients, such as VB_1_, in brown rice, while increasing the levels of dietary fiber, total phenols, and VE. This phenomenon may be attributed to the structural alterations in starch and starch–lipid complex and the diffusion of moisture induced by the treatment [36,37]. These changes contribute to a rise in resistant starch content and the migration of other nutritional factors [38], ultimately exerting beneficial effects on the overall nutritional profile of brown rice.

### 3.2. Appearance and Texture Analysis

The thicknesses, diameters, and spread ratios of the three types of biscuits were 5.56–10.50 mm, 42.63–49.40 mm, and 4.06–8.89, respectively (Table 2). Compared with the control (100% wheat flour biscuits), the thicknesses of BRB and HBRB decreased, and the diameter and spread ratio increased. The maximum spread ratio of the HMT biscuits was 8.89, which was much higher than that of the control (4.06). The spread ratio is an important characteristic determining biscuit quality, which is closely related to the viscosity of the dough [39]. The observed result may be explained by two factors. Firstly, the high dietary fiber in BRB and HBRB competes with starch for water, thereby inhibiting starch gelatinization, limiting dough expansion, and ultimately increasing the biscuit spread ratio [40,41]. Secondly, HMT induces changes in the rearrangement of starch and protein molecules [7], which alters starch gelatinization properties, reduces dough viscosity, and thus further promotes spread ratio.

The hardness and brittleness of the biscuits are listed in Table 2. The hardness of the control biscuit was 700.82 g and the brittleness 1068.89 g/sec, whereas the corresponding values for the BRB samples were 1177.53 g and 1973.20 g/sec and for the HBRB samples, 1085.91 g and 2067.18 g/sec, respectively. HMT significantly enhanced both the hardness and brittleness of the biscuits, a finding consistent with that reported by Yang et al. [5]. This textural improvement can be attributed to two main mechanisms. Firstly, the increase in dietary fiber content resulting from the HMT plays a critical role. Cakır et al., who demonstrated that the incorporation of fiber-rich plums into rice flour significantly increased the hardness of gluten-free cakes [42]. Secondly, it may be because HMT promotes starch retrogradation in brown rice biscuits during cooling [43].

Color is the primary parameter determining the initial acceptance of baked products [44]. It can be seen that the main changes of different biscuits are reflected in the values of a* and L* in Table 2. The L* and a* values of different biscuits ranged from 75.07 to 81.46 and from 1.75 to 3.04, respectively. The control had the highest L* value (81.46) and the lowest a* value (1.75). In contrast, the HBRB had an L* value of 77.54 and an a* value of 2.91, its L* value was significantly lower, and its a* value was significantly higher than those of the control (*p* < 0.05), indicating that the control had the highest brightness and lowest redness of the biscuits, which may have been due to the raw materials. This result may be related to the raw materials’ color. Wheat flour is bright in color, whereas brown rice flour exhibits a reddish hue. Furthermore, brown rice bran contains dietary fiber, total phenolics, and other compounds which participate in the Maillard reaction during baking, ultimately resulting in the lower L* value and higher a* value observed in BRB. After HMT, the browning of brown rice under high temperatures, caused by covalent bonding between reducing sugars and proteins, contributes to a red color shift [45], thereby resulting in significant differences in the L* and a* values of HBRB.

### 3.3. Microstructure Analysis

**Figure 1:**
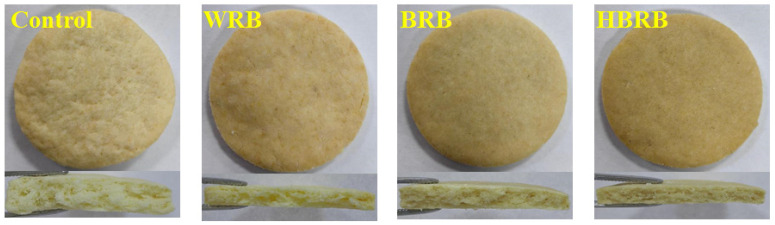
Surface and cross-section of biscuits.

**Figure 2:**
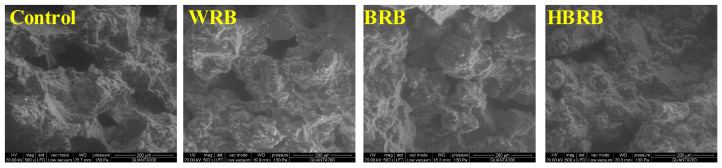
Scanning electron microscopy (SEM) images of biscuits, magnification ×500.

The surface, cross-sectional image, and microstructure of the biscuits are shown in Figure 1 and Figure 2. As seen in Figure 1, the control sample exhibited an uneven surface with irregular pores, followed by WRB, which showed a relatively smooth surface but a greater number of cracks. In contrast, both BRB and HBRB displayed better appearance, in particular, HBRB presented a smooth and delicate surface, albeit with the darkest color, which is consistent with the chromaticity value. SEM results (Figure 2) revealed that the starch gelatinized to form a gel state during the baking process, so the clustered starch particles and tightly wrapped structure of the protein matrix are shown in the cross-section [30]. Wheat flour contains a large amount of gluten that forms a three-dimensional structure during baking, resulting in more pores and lower hardness. In contrast, the HMT modified the structure of components such as dietary fiber, starch, and protein in brown rice, ultimately contributing to a better organized biscuit structure.

### 3.4. Sensory Evaluation of Biscuits

**Figure 3:**
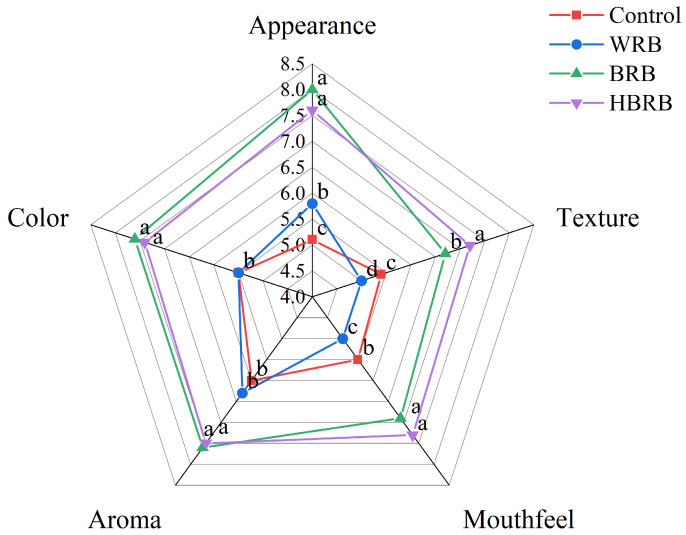
Sensory evaluation results of the biscuits. Values in the same row followed by different superscript letters are significant at *p* < 0.05.

Figure 3 displays the sensory evaluation results of different biscuits. BRB achieved the highest score in appearance (8.0), followed by HBRB (7.6), which may be attributed to the increased dietary fiber content enhancing the biscuit spread ratio. A similar trend was observed in color scores: the control group and WBR received the lowest color scores (5.5), while BRB obtained the highest (7.6), followed by HBRB (7.4). This may be due to the polyphenols, dietary fiber, and other components in brown rice promoting enzymatic browning during high-temperature baking. However, the HMT deepened the color of brown rice, thereby influencing the color score. HMT positively affected the texture and taste of the biscuits. HBRB received the highest scores for texture and mouthfeel, at 7.2 and 7.3, respectively, which may be related to the HMT promoting the formation of a denser structure in the biscuits [46]. This result was also consistent with the microstructure analysis. In terms of flavor, baking facilitated the release of flavor compounds from brown rice [47], making the biscuits more preferred by consumers. The sensory evaluation results highlight the potential of using heat moisture treated brown rice in biscuit production: to enhance the product’s sensory quality and to promote the comprehensive development of whole grains.

### 3.5. Storage Characteristics of Biscuits

**Figure 4:**
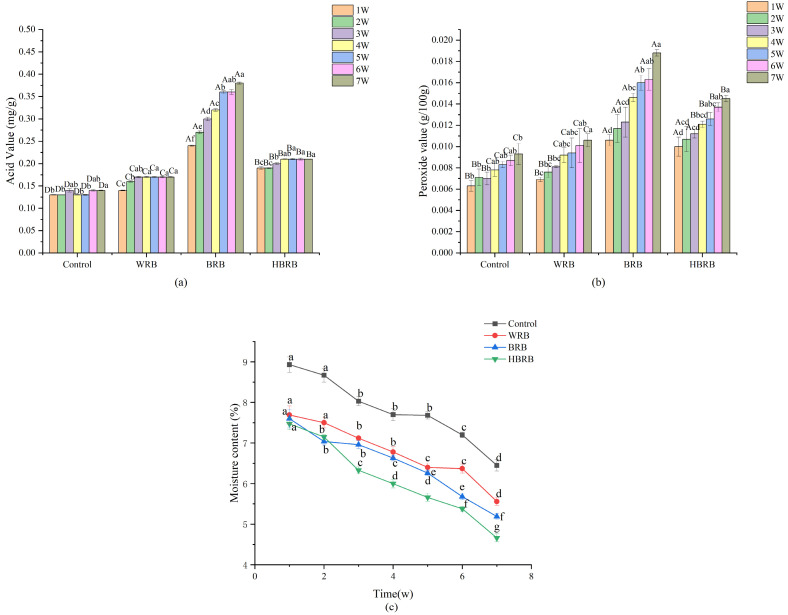
Storage properties of the biscuits: (**a**) acid value, (**b**) peroxide value, and (**c**) moisture content. Lowercase letters (a, b, c, etc.) indicate significant differences (*p* < 0.05) in acid value and peroxide value of the same biscuit sample under different storage periods; Uppercase letters (A, B, C, etc.) indicate significant differences (*p* < 0.05) in acid value and peroxide value among different biscuit samples under the same storage period.

The moisture content of the biscuits decreased gradually with increasing storage time. Compared with the control, the WRB, BRB, and HBRB groups lost more moisture, and the moisture loss of the HBRB group was the highest at 38%. This may be attributed to the higher dietary fiber content in heat moisture treated brown rice, which inhibited starch from absorbing water during gelatinization, consequently leading to increased moisture loss in the biscuits during storage (Figure 4c).

Free fatty acid and peroxide values are important indicators of quality changes during biscuit storage, reflecting the degree of oxidation of fats and fatty acids in biscuits [33]. As shown in Figure 4a,b, the free fatty acid and peroxide values of the four biscuit samples showed a gradually increasing trend with the extension of storage time. In contrast, BRB had a higher degree of oil oxidation because of the fat content in the bran compared to the control and WRB. In Figure 4a, the variation range of free fatty acids of the four groups of biscuits were 0.13–0.14 mg/g, 0.14–0.17 mg/g, 0.24–0.38 mg/g, 0.19–0.21 mg/g, respectively. The free fatty acids of BRB had the largest variation range and the highest value during storage, whereas the corresponding values in HBRB, although higher than those of the control and WRB, were significantly lower than those of BRB (*p* < 0.05). This may be because HMT passivates the lipase in brown rice bran, inhibits their activity, and weakens the ability of fatty acids to be broken down by lipases during storage [48]. The peroxide value of HBRB was also significantly lower than that of the BRB group. By contrast, BRB exhibited significantly higher peroxide and acid values throughout the storage period. This indicates that both the generation rate and accumulation of free fatty acids and peroxides were greater in BRB than in HBRB. These findings further indicate that heat moisture treatment delayed the oxidation process of brown rice biscuits.

## 4. Conclusions

Gluten-free biscuits prepared from brown rice subjected to a combined treatment of HMT and ultrafine grinding showed an improved texture and enhanced sensory quality. Furthermore, HMT effectively improved the storage stability of the biscuits, supporting the utilization of brown rice as a whole grain. While our findings demonstrate a clear association between the combined treatment and improved brown rice biscuit quality, the underlying mechanisms remain incompletely explored. Therefore, elucidating the interactions among the major components (starch, protein, lipid) and their impact on digestibility and glycemic index (GI) represents a critical next step for enhancing the application value of this research.

## Figures and Tables

**Table 1:** Nutritional components of biscuits.

Index	Control	WRB	BRB	HBRB
Protein %	8.83 ± 0.12 ^a^	7.75 ± 0.11 ^c^	8.44 ± 0.10 ^b^	8.60 ± 0.15 ^ab^
Fat %	14.73 ± 0.14 ^b^	14.22 ± 0.19 ^b^	16.51 ± 0.23 ^a^	16.59 ± 0.23 ^a^
Carbohydrate %	62.98 ± 0.45 ^b^	67.26 ± 0.53 ^a^	62.02 ± 0.44 ^bc^	61.49 ± 0.15 ^c^
Moisture %	8.93 ± 0.19 ^a^	7.69 ± 0.22 ^b^	7.60 ± 0.23 ^b^	7.47 ± 0.14 ^b^
Total dietary fiber %	2.67 ± 0.19 ^b^	1.27 ± 0.20 ^c^	3.27 ± 0.12 ^a^	3.62 ± 0.15 ^a^
VB_2_ mg/100 g	--	--	0.069 ± 0.0017	0.065 ± 0.0016
Total phenolics %	0.053 ± 0.003 ^d^	0.094 ± 0.003 ^c^	0.127 ± 0.009 ^b^	0.154 ± 0.007 ^a^
VB_1_ mg/100 g	--	--	0.179 ± 0.013	0.126 ± 0.008
VE mg/100 g	0.574 ± 0.009 ^c^	0.588 ± 0.015 ^c^	0.965 ± 0.017 ^b^	1.483 ± 0.035 ^a^
Ash %	1.86 ± 0.10 ^b^	1.82 ± 0.12 ^b^	2.16 ± 0.13 ^a^	2.23 ± 0.10 ^a^

Note: The data is expressed as mean ± standard deviation (*n* = 3). Values in the same row followed by different superscript letters are significant at *p* < 0.05. “--” representative not detected.

**Table 2:** The physical and color properties of biscuits.

Properties	Index	Control	WRB	BRB	HBRB
Physical analysis	Thickness/mm	10.50 ± 0.23 ^a^	6.26 ± 0.22 ^b^	6.11 ± 0.19 ^b^	5.56 ± 0.10 ^c^
	Diameter/mm	42.63 ± 1.01 ^c^	46.20 ± 0.89 ^b^	48.00 ± 0.36 ^ab^	49.40 ± 0.36 ^a^
	Spread ratio	4.06 ± 0.18 ^c^	7.40 ± 0.41 ^b^	7.86 ± 0.28 ^b^	8.89 ± 0.22 ^a^
	Hardness/g	700.82 ± 11.88 ^c^	921.30 ± 17.62 ^b^	1177.53 ± 39.86 ^a^	1085.91 ± 58.06 ^a^
	Brittleness/g/sec	1068.89 ± 53.55 ^c^	1593.67 ± 39.35 ^b^	1973.20 ± 29.67 ^a^	2067.18 ± 52.76 ^a^
Color parameters	a* (redness)	1.75 ± 0.39 ^b^	2.45 ± 2.47 ^ab^	3.04 ± 0.39 ^a^	2.91 ± 0.54 ^a^
	b* (yellowness)	29.15 ± 0.35 ^a^	29.96 ± 0.57 ^a^	29.38 ± 0.34 ^a^	29.47 ± 0.75 ^a^
	L* (lightness)	81.46 ± 0.53 ^a^	79.58 ± 0.49 ^b^	76.44 ± 0.36 ^c^	75.07 ± 0.54 ^d^
	∆E	--	1.80 ± 0.25 ^c^	4.73 ± 0.31 ^b^	5.91 ± 0.04 ^a^

Note: The data is expressed as mean ± standard deviation (*n* = 3). Values in the same row followed by different superscript letters are significant at *p* < 0.05.

## Data Availability

The original contributions presented in the study are included in the article, further inquiries can be directed to the corresponding author.

## References

[1] Anggraeni A.A., Triwitono P., Lestari L.A., Harmayani E. (2024). Evaluation of glucomannan as a fat replacer in the dough and cookies made from fermented cassava flour and soy protein concentrate. Food Chem..

[2] Benanti A., Rabie Ashkezary M., Gugino I.M., Canale M., Yeganehzad S., Todaro A. (2023). Evaluation of biscuits obtained from novel composite flour containing Maiorca malt flour. Ital. J. Food Sci..

[3] Sharma N., Bhatia S., Chunduri V., Kaur S., Sharma S., Kapoor P., Kumari A., Garg M. (2020). Pathogenesis of Celiac Disease and Other Gluten Related Disorders in Wheat and Strategies for Mitigating Them. Front. Nutr..

[4] Schmelter L., Rohm H., Struck S. (2021). Gluten-free bakery products: Cookies made from different Vicia faba bean varieties. Future Foods.

[5] Yang L., Wang S., Li S., Zhao G., Du C. (2022). Effect of Heat-Moisture Treatment on the Physicochemical Properties and Starch Digestibility of Mix Powder (Wheat Flour-Black Soybean Flour) and Corresponding Cookies. Gels.

[6] Cheng Z., Li N., Chen Z., Li K., Qiao D., Zhao S., Zhang B. (2023). Ingesting retrograded rice (*Oryza sativa*) starch relieves high-fat diet induced hyperlipidemia in mice by altering intestinal bacteria. Food Chem..

[7] Guo Y., Fang R., Wu Z., Xi G., Qiao D., Wang G., Cui T., Zhang L., Zhao S., Zhang B. (2024). Incorporating edible oil during cooking tailors the microstructure and quality features of brown rice following heat moisture treatment. Food Res. Int..

[8] Saleh A.S.M., Wang P., Wang N., Yang L., Xiao Z. (2019). Brown Rice Versus White Rice: Nutritional Quality, Potential Health Benefits, Development of Food Products, and Preservation Technologies. Compr. Rev. Food Sci. Food Saf..

[9] Pletsch E.A., Hamaker B.R. (2018). Brown rice compared to white rice slows gastric emptying in humans. Eur. J. Clin. Nutr..

[10] Liu Y.Q., Strappe P., Zhou Z.K., Blanchard C. (2019). Impact on the nutritional attributes of rice bran following various stabilization procedures. Crit. Rev. Food Sci. Nutr..

[11] Kalahal S.P., Gavahian M., Lin J. (2024). Development of innovative tigernut-based nutritional snack by extrusion process: Effects of die temperature, screw speed, and formulation on physicochemical characteristics. Qual. Assur. Saf. Crops Foods.

[12] Wei S., Wang N., Huang X., Xu G., Xu X., Xu D., Jin Y., Yang N., Wu F. (2022). Effect of germination on the quality characteristics and volatile compounds of fermented brown rice cake. Food Biosci..

[13] Erinc H., Mert B., Tekin A. (2018). Different sized wheat bran fibers as fat mimetic in biscuits: Its effects on dough rheology and biscuit quality. J. Food Sci. Technol..

[14] Zhao X., Yang Z., Gai G., Yang Y. (2009). Effect of superfine grinding on properties of ginger powder. J. Food Eng..

[15] Muttakin S., Kim M.S., Lee D.-U. (2015). Tailoring physicochemical and sensorial properties of defatted soybean flour using jet-milling technology. Food Chem..

[16] Lin S., Gao J., Jin X., Wang Y., Dong Z., Ying J., Zhou W. (2020). Whole-wheat flour particle size influences dough properties, bread structure and in vitro starch digestibility. Food Funct..

[17] Klein B., Pinto V.Z., Vanier N.L., Zavareze E.d.R., Colussi R., Evangelho J.A.d., Gutkoski L.C., Dias A.R.G. (2013). Effect of single and dual heat–moisture treatments on properties of rice, cassava, and pinhao starches. Carbohydr. Polym..

[18] Xie X., Qi L., Xu C., Shen Y., Wang H., Zhang H. (2020). Understanding how the cooking methods affected structures and digestibility of native and heat-moisture treated rice starches. J. Cereal Sci..

[19] Aluthge D.S.U., Ranaweera K.K.D.S., Gunathilake I.A.D.S.R. (2023). The effect of stabilization heat treatment on rice bran quality parameters, including total phenolic content, gamma oryzanol content, antioxidant potential, oxidative stability and extraction yield during storage. Food Chem. Adv..

[20] Wei Q., Guo Y., Liu X., Wang S., Xu Z., Chen S. (2022). Improving the eating quality of brown rice by defatting combined with hydrothermal treatment. Food Res. Int..

[21] Liu Y., Jia Z., Li M., Bian K., Guan E., Huang W. (2024). Effect of heat-moisture treatment of wheat (Triticum aestivum L.) grain on micronutrient content of flour, and noodles and bread qualities. J. Cereal Sci..

[22] Zhang G., Xuan Y., Lyu F., Ding Y. (2023). Microstructural, physicochemical properties and starch digestibility of brown rice flour treated with extrusion and heat moisture. Int. J. Biol. Macromol..

[23] Islam M.Z., Taneya M.L.J., Shams-Ud-Din M., Syduzzaman M., Hoque M.M. (2012). Physicochemical and Functional Properties of Brown Rice (*Oryza sativa*) and Wheat (*Triticum aestivum*) Flour and Quality of Composite Biscuit Made Thereof. Agriculturists.

[24] Horwitz W., Latimer G., AOAC (2016). AOAC (association of official agricultural chemists). The Official Methods of Analysis of AOAC International.

[25] Biswas R., Sarkar A., Alam M., Roy M., Mahdi Hasan M.M. (2023). Microwave and ultrasound-assisted extraction of bioactive compounds from Papaya: A sustainable green process. Ultrason. Sonochem..

[26] Norhayati M.K., Fairulnizal M.N.M., Zaiton A., Syuriahti W.Z.W., Rusidah S., Aswir A.R., Ang J.L., Naeem M.N.M., Suraiami M., Azerulazree J.M. (2015). Nutritional Composition of Selected Commercial Biscuits in Malaysia. Sains Malays..

[27] AACC (2016). American Association for Cereal Chemistry.

[28] Sulieman A.A., Zhu K.-X., Peng W., Hassan H.A., Obadi M., Siddeeg A., Zhou H.-M. (2019). Rheological and quality characteristics of composite gluten-free dough and biscuits supplemented with fermented and unfermented Agaricus bisporus polysaccharide flour. Food Chem..

[29] Dey B.C., Abedin M.Z., Haque M.A., Molla M.M., Alam M., Bari L., Zubair M.A., Sikder M.A. (2025). Nutritional profile and bioactive potential of Ivy gourd (*Coccinia grandis* L. Voigt) fruit and quality evaluation of its developed biscuits. Food Chem. Adv..

[30] Yang L., Wang S., Zhang W., Zhang H., Guo L., Zheng S., Du C. (2022). Effect of black soybean flour particle size on the nutritional, texture and physicochemical characteristics of cookies. LWT.

[31] Sensory Analysis—Methodology—General Guidance for Conducting Hedonic Tests with Consumers in a Controlled Area. https://www.iso.org/standard/50125.html.

[32] Wang X., Lu L., Hayat K., Xia S. (2024). Effect of chickpea thermal treatments on the starch digestibility of the fortified biscuits. Food Biosci..

[33] Kumar P.K.P., Manohar R.S., Indiramma A.R., Krishna A.G.G. (2014). Stability of oryzanol fortified biscuits on storage. J. Food Sci. Technol..

[34] Malekian F. (2000). Lipase and lipoxygenase activity, functionality, and nutrient losses in rice bran during storage. LSU AgCenter.

[35] Rashid N.Y.A., Razak D.L.A., Jamaluddin A., Sharifuddin S.A., Long K. (2015). Bioactive compounds and antioxidant activity of rice bran fermented with lactic acid bacteria. Malays. J. Microbiol..

[36] Lin Z., Zhang R., Wu Z., Qiao D., Zhao S., Pi X., Zhang B. (2024). Prolonging heat-moisture treatment time at medium moisture content optimizes the quality attributes of cooked brown rice through starch structural alteration. Int. J. Biol. Macromol..

[37] Balbinoti T.C.V., Jorge L.M.d.M., Jorge R.M.M. (2018). Modeling the hydration step of the rice (*Oryza sativa*) parboiling process. J. Food Eng..

[38] Zhang S., Ma Y., Ren X., Wu W., Liao L. (2025). Combined Lactobacillus plantarum fermentation and heat-moisture treatment: Correlation analysis of physicochemical properties of rice flour and quality of rice noodles. Int. J. Gastron. Food Sci..

[39] Pareyt B., Talhaoui F., Kerckhofs G., Brijs K., Goesaert H., Wevers M., Delcour J.A. (2009). The role of sugar and fat in sugar-snap cookies: Structural and textural properties. J. Food Eng..

[40] Gómez M., Zhou W., Gao J. (2022). Chapter Five—Gluten-free bakery products: Ingredients and processes. Advances in Food and Nutrition Research.

[41] Paesani C., Bravo-Núñez Á., Gómez M. (2020). Effect of extrusion of whole-grain maize flour on the characteristics of gluten-free cookies. LWT.

[42] Cakir E., Ozülkü G., Bekiroglu H., Arici M., Sagdic O. (2024). Technological quality, bioactive features, and glycemic index of gluten-free cakes formulated with lyophilized wild Prunus spinosa fruit. Qual. Assur. Saf. Crops Foods.

[43] Chung H.-J., Cho A., Lim S.-T. (2012). Effect of heat-moisture treatment for utilization of germinated brown rice in wheat noodle. LWT.

[44] Culetu A., Stoica-Guzun A., Duta D.E. (2021). Impact of fat types on the rheological and textural properties of gluten-free oat dough and cookie. Int. J. Food Sci. Technol..

[45] Thuengtung S., Ketnawa S., Ding Y., Cai Y., Ogawa Y. (2023). Effect of mild heat-moisture treatment for harvested raw paddy rice on physicochemical properties and in vitro starch digestibility of cooked rice. Food Hydrocoll. Hlth..

[46] Chung H.-J., Cho A., Lim S.-T. (2014). Utilization of germinated and heat-moisture treated brown rices in sugar-snap cookies. LWT.

[47] Sun Z., Lyu Q., Chen L., Zhuang K., Wang G., Ding W., Wang Y., Chen X. (2022). An HS-GC-IMS analysis of volatile flavor compounds in brown rice flour and brown rice noodles produced using different methods. LWT.

[48] Nakamura S., Okumura H., Sugawara M., Noro W., Homma N., Ohtsubo K. (2017). Effects of different heat–moisture treatments on the physicochemical properties of brown rice flour. Biosci. Biotechnol. Biochem..

